# Cardiac Troponin I and Cardiovascular Risk in Patients With Chronic Obstructive Pulmonary Disease

**DOI:** 10.1016/j.jacc.2018.06.051

**Published:** 2018-09-04

**Authors:** Philip D. Adamson, Julie A. Anderson, Robert D. Brook, Peter M.A. Calverley, Bartolome R. Celli, Nicholas J. Cowans, Courtney Crim, Ian J. Dixon, Fernando J. Martinez, David E. Newby, Jørgen Vestbo, Julie C. Yates, Nicholas L. Mills

**Affiliations:** a British Heart Foundation Centre for Cardiovascular Science, University of Edinburgh, Edinburgh, United Kingdom; b Research & Development, GSK, Stockley Park, Middlesex, United Kingdom; c Division of Cardiovascular Medicine, University of Michigan, Ann Arbor, Michigan; d Department of Medicine, Clinical Sciences Centre, University of Liverpool, University Hospital Aintree, Liverpool, United Kingdom; e Pulmonary and Critical Care Division, Brigham and Women’s Hospital, Harvard Medical School, Boston, Massachusetts; f Statistics and Programming, Veramed, Twickenham, United Kingdom; g Research & Development, GSK, Research Triangle Park, North Carolina; h Joan and Sanford I. Weill Department of Medicine, Weill Cornell Medicine, New York, New York; i Division of Infection, Immunity and Centre for Respiratory Medicine and Allergy, Manchester Academic Health Science Centre, The University of Manchester and Manchester University NHS Foundation Trust, Manchester, United Kingdom

**Keywords:** cardiac troponin, cardiovascular risk, chronic obstructive pulmonary disease, CI, confidence interval, COPD, chronic obstructive pulmonary disease, CV, cardiovascular, FEV_1,_, forced expiratory volume in 1 s, HR, hazard ratio, ICS, inhaled corticosteroid, IQR, interquartile range, LABA, long-acting beta-agonist

## Abstract

**Background:**

Patients with chronic obstructive pulmonary disease (COPD) have increased risk of cardiovascular events.

**Objectives:**

This study evaluated the association between high-sensitivity cardiac troponin I concentration and cardiovascular events in patients with COPD and heightened cardiovascular risk.

**Methods:**

In a double-blind randomized controlled trial, 16,485 patients with COPD and cardiovascular disease or risk factors were randomized to once daily inhaled placebo, fluticasone furoate (100 μg), vilanterol (25 μg), or their combination. Plasma high-sensitivity cardiac troponin I concentrations were measured in a subgroup of 1,599 patients. Outcomes were on-treatment cardiovascular events and COPD exacerbations over a median of 18 months, and cardiovascular death over a median of 27 months.

**Results:**

Baseline plasma cardiac troponin I concentrations were above the limit of detection (1.2 ng/l) in 1,542 (96%) patients. Concentrations were unaffected by inhaled therapies at 3 months (p > 0.05). Compared with the lowest quintile (cardiac troponin <2.3 ng/l), patients in the highest quintile (≥7.7 ng/l) were at greater risk of cardiovascular events (hazard ratio [HR] 3.7; 95% confidence interval [CI]: 1.3 to 10.1; p = 0.012) and cardiovascular death (HR: 20.1; 95% CI: 2.4 to 165.2; p = 0.005) after adjustment for risk factors. By contrast, there were no differences in exacerbations between quintiles (HR: 1.1; 95% CI: 0.8 to 1.5; p = 0.548).

**Conclusions:**

In patients with COPD and heightened cardiovascular risk, plasma cardiac troponin I concentrations are a specific and major indicator of future cardiovascular events and cardiovascular death. Inhaled therapies did not affect cardiac troponin I concentrations consistent with their neutral effect on mortality and cardiovascular outcomes. (Study to Evaluate the Effect of Fluticasone Furoate/Vilanterol on Survival in Subjects With Chronic Obstructive Pulmonary Disease [SUMMIT]; NCT01313676)

Cardiovascular (CV) disease, including ischemic heart disease and stroke, accounts for 1 in 4 deaths globally and is increasing in prevalence (1). Despite recent advances in understanding risk factors and therapeutic interventions, atherosclerotic events remain unacceptably common. Residual risk is particularly high among patients with proinflammatory comorbidities, such as chronic obstructive pulmonary disease (COPD) (2). In some cases, it remains unclear whether it is the disease process itself, or the off-target effects of the pharmacological treatments, that contribute to this elevated risk (3).

Notwithstanding prior major advances, future clinical trial conduct is hampered by several important and increasing challenges. It is well recognized that clinical trial participants represent a relatively low-risk subset of the real-world patient population. Consequently, modest event rates necessitate large and costly trials in order to demonstrate treatment efficacy. Conversely, this low event rate creates the potential for researchers to fail to recognize CV harms related to new medications before their clinical approval (4). These challenges have contributed to growing interest in the search for better biomarkers suitable for use as a surrogate for treatment efficacy and safety. Such tests could provide an indication of risk-benefit balance in earlier-phase clinical trials and may better inform the design of large-scale clinical endpoint trials.

An ideal CV biomarker needs to be a sensitive as well as a specific indicator of CV risk. High-sensitivity cardiac troponin I is such a potential suitable candidate. Plasma concentrations can be reliably quantified in most apparently healthy individuals, and numerous studies have demonstrated clear associations between elevated plasma troponin concentrations and CV events in both primary and secondary prevention populations 5, 6, 7, 8, 9, 10, 11. Furthermore, plasma cardiac troponin I concentrations measured by a high-sensitivity assay have recently been shown to be modifiable, with statin-induced reductions in cardiac troponin I proving a more powerful indicator of treatment efficacy than changes in serum cholesterol (12). The role of serial testing with high-sensitivity cardiac troponin I to predict the effect of other therapies on CV outcomes has to date been unexplored in patients with more diverse multimorbid conditions.

SUMMIT (Study to Understand Mortality and MorbidITy) 13, 14 assessed the efficacy and safety of inhaled corticosteroids and long-acting beta-agonists (LABAs) in 16,485 patients with COPD and heightened CV risk. This was a multimorbid population with interventions that could have both benefit (15) and harm (16). The present study reports post hoc analyses aiming to determine whether plasma high-sensitivity cardiac troponin I concentrations can stratify CV risk, be modified by inhaled corticosteroids (ICS) and bronchodilators, and predict outcomes within the context of SUMMIT.

## Methods

### Study population

The prospective, multicenter, international randomized controlled SUMMIT trial sought to determine whether treatment with an inhaled LABA in combination with an ICS versus either component, could improve clinical outcomes in patients with moderate COPD and increased CV risk compared with placebo. Details regarding study design have been previously published 13, 14. In brief, eligible participants included current or former smokers (≥10 pack-years) between the ages of 40 and 80 years, with a history of COPD and a post-bronchodilator forced expiratory volume in 1 s (FEV_1_) ≥50% and ≤70% of the predicted value, a ratio of post-bronchodilator FEV_1_ to forced vital capacity ≤0.70, and a score ≥2 on the modified Medical Research Council dyspnea scale. Patients were additionally required to have a history, or be at increased risk, of CV disease. CV disease was defined as coronary artery disease, peripheral arterial disease, prior stroke or myocardial infarction, or diabetes mellitus with target organ disease. Increased CV risk was defined as being ≥60 years and receiving medications for ≥2 of the following: hypercholesterolemia, hypertension, diabetes mellitus, or peripheral vascular disease.

Although prior ICS and LABA treatments were discontinued before study entry, other COPD medications were permitted during the trial. Participants were then allocated equally to 1 of 4 randomized treatments: placebo, fluticasone furoate (100 μg), vilanterol (25 μg), or their combination (fluticasone furoate/vilanterol, 100/25 μg) inhaled once daily as a dry powder. A total of 16,485 patients were enrolled and included in the final intention-to-treat efficacy population.

### Endpoints

In addition to the primary endpoint of all-cause mortality by intention-to-treat analysis, the secondary CV endpoint was time to first-on-treatment CV event comprising CV death, myocardial infarction, stroke, unstable angina, and transient ischemic attack (17). Categorization of the cause of each death was adjudicated by a clinical endpoint committee blinded to the treatment allocation who also determined whether any reported CV event met the definition of the composite endpoint (13). Individuals discontinuing study treatments could not be assessed for the adjudicated composite CV endpoint because follow-up visits were not performed, and only data regarding mortality were available. Another endpoint comprised moderate or severe exacerbations of COPD. Moderate exacerbations were defined as a symptomatic deterioration requiring treatment with antibiotic drugs or systemic corticosteroids, whereas severe exacerbations were defined as events leading to hospital admission.

### High-sensitivity cardiac troponin I

Venous blood samples were obtained before randomization and at 3 months. Blood was processed and plasma stored at −80°C until analyzed. As previously described, before analysis, samples were thawed and underwent centrifugation twice (3,000 relative centrifugal force for 10 min) according to the manufacturer’s instructions to ensure homogeneity (12). Plasma high-sensitivity cardiac troponin I concentrations were measured at a single site using the ARCHITECT_*STAT*_ high-sensitive cardiac troponin I assay (Abbott Laboratories, Abbott Park, Illinois), which has a limit of detection of 1.2 ng/l, coefficient of variation <10% at 4.7 ng/l, and sex-specific 99th percentile upper reference limits of 16 and 34 ng/l in women and men, respectively 18, 19.

### Statistical analysis

A value of 0.5 ng/l was imputed for patients without reportable troponin values. Cardiac troponin I concentrations were log-transformed before statistical modeling, and results transformed back to the original scale. To determine which patient characteristics were associated with baseline cardiac troponin I, regression modeling was performed. The final model was achieved using backwards selection where to remain in the model all variables needed to have p < 0.10. To test whether ICS or LABA therapy affected cardiac troponin I values at 3 months, an analysis of covariance was performed adjusting for baseline cardiac troponin I, age, sex, prior myocardial infarction and hypertension.

Patients were grouped into quintiles based on their baseline cardiac troponin I concentrations. To explore the effect of baseline cardiac troponin I quintile on each of the study endpoints (CV composite, CV death, and COPD exacerbations), analysis of time-to-first event was performed using Cox proportional hazards regression modeling, adjusted for age, sex, study therapy, and CV risk factors of prior myocardial infarction and hypertension. In a sensitivity analysis, we also included statin therapy and C-reactive protein concentrations as model covariates. Cardiac troponin I was also examined as a continuous variable where the best fitting model was selected from a variety of polynomial or logarithmic models using 2-term fractional polynomials (20).

Previous reports have identified that adverse CV outcomes are associated with plasma troponin I concentrations ≥5 ng/l 12, 21. To explore this association further, and investigate whether the predictive value of this threshold could be applied to the SUMMIT population, patients were grouped into those who had plasma concentrations <5 ng/l at both baseline and 3 months, and those with a concentration ≥5 ng/l at either baseline or 3 months.

Scientific oversight of the trial was provided by a steering committee composed of academic experts and employees from GlaxoSmithKline, who were collectively responsible for the study design and analysis, and for the review and interpretation of the data. This study is registered with ClinicalTrials.gov (NCT01313676).

## Results

The study population and principal findings of the SUMMIT study have previously been described (14). Between January 2011 and March 2014, 16,485 participants were recruited and included in the primary intention-to-treat analysis (17). Blood samples were taken before randomization from 1,673 patients based in the United States (SUMMIT biomarker population), of which baseline cardiac troponin I concentrations were assessed in 1,599 patients, and 1,258 had a second troponin measurement performed 3 months after randomization. The majority of patients included in this analysis had established CV disease or diabetes mellitus with end-organ damage (n = 1,163 [73%]), whereas a minority (n = 407 [25%]) fulfilled the criteria for an increased risk of CV disease only, and 29 (2%) did not meet the CV entry criteria.

### Distribution of high-sensitivity cardiac troponin I concentrations at baseline

Cardiac troponin I concentrations were ≥1.2 ng/l in 1,542 participants (96%) and above the sex-specific 99th percentile (16 ng/l in women, 34 ng/l in men) in 42 participants (2.6%). The median cardiac troponin I concentration was 4.0 ng/l (interquartile range [IQR]: 2.6 to 6.7 ng/l).

The patient characteristics in the biomarker substudy population were broadly similar to the overall SUMMIT population, except that those in the biomarker population were more likely to be female, have a higher body mass index, have fewer previous COPD exacerbations, and have differences in CV history and CV therapy (Table 1). Participants were stratified into quintiles by plasma cardiac troponin I concentration from samples obtained before randomization. Compared with the lowest quintile (<2.3 ng/l), patients in the highest quintile (≥7.7 ng/l) were older, more likely to be male, former smokers, have higher systolic blood pressure, a history of ischemic heart disease, coronary artery disease, congestive heart failure, hypercholesterolemia, hypertension, diabetes mellitus, a family history of myocardial infarction or stroke, and to be receiving treatment with antiplatelet and statin therapies.

**Table 1:** Patient Characteristics in the SUMMIT Study Population, the Biomarker Substudy Population, and Split by Cardiac Troponin I Quintile

	Troponin Quintile 1 (<2.3 ng/l) (n = 307)	Troponin Quintile 2 (≥2.3 to <3.4 ng/l) (n = 32)	Troponin Quintile 3 (≥3.4 to <4.8 ng/l) (n = 31)	Troponin Quintile 4 (≥4.8 to <7.7 ng/l) (n = 33)	Troponin Quintile 5 (≥7.7 ng/l) (n = 31)	Biomarker Substudy∗ (n = 1,67)	SUMMIT ITT-E Population (n = 16,48)
Median troponin	1.7	2.8	4.0	5.8	12.0	4.0	-
Age, yrs	63 ± 8	65 ± 8	67 ± 8	68 ± 7	68 ± 7	66 ± 8	65 ± 8
Female	172 (56)	153 (47)	107 (34)	98 (30)	80 (25)	635 (38)	4,196 (25)
BMI, kg/m^2^	30 ± 6	31 ± 7	31 ± 6	31 ± 7	31 ± 7	31 ± 7	28 ± 6
Systolic blood pressure, mm Hg	128 ± 14	129 ± 16	132 ± 16	131 ± 16	134 ± 19	131 ± 16	135 ± 15
Heart rate, beats/min	75 ± 10	74 ± 11	73 ± 11	73 ± 11	73 ± 11	73 ± 11	76 ± 10
Estimated GFR, ml/min/1.73 m^2^	101.2 ± 33.6	100.5 ± 37.1	100.5 ± 38.4	95.2 ± 36.1	89.3 ± 34.9	97.7 ± 36.7	97.3 ± 36.6
CRP, mg/l	5.3 ± 6.9	6.5 ± 8.3	5.6 ± 7.0	6.5 ± 10.3	6.8 ± 8.7	6.2 ± 8.3	6.2 ± 8.3
Past medical history							
Prior myocardial infarction or coronary revascularization	71 (23)	89 (27)	112 (35)	145 (44)	162 (51)	601 (36)	3,436 (21)
Coronary artery disease	113 (37)	132 (41)	148 (46)	186 (56)	199 (63)	818 (49)	8,379 (51)
Congestive heart failure	15 (5)	11 (3)	21 (7)	34 (10)	61 (19)	146 (9)	3,456 (21)
Hypercholesterolemia	243 (79)	280 (86)	283 (89)	295 (89)	292 (92)	1458 (87)	11,518 (70)
Hypertension	258 (84)	285 (88)	300 (94)	307 (93)	302 (95)	1519 (91)	14,851 (90)
Diabetes mellitus	108 (35)	116 (36)	116 (36)	138 (42)	140 (44)	642 (38)	4,997 (30)
Family history of CVD	128 (42)	128 (39)	116 (36)	146 (44)	145 (46)	691 (41)	3,429 (21)
Respiratory history							
Former smoker	141 (46)	152 (47)	170 (53)	178 (54)	169 (53)	845 (51)	8,807 (53)
Post-bronchodilator FEV_1_, l	1.7 ± 0.4	1.7 ± 0.4	1.7 ± 0.4	1.7 ± 0.4	1.7 ± 0.4	1.7 ± 0.4	1.7 ± 0.4
Predicted post-bronchodilator FEV_1_, % of predicted	59.7 ± 6.9	59.4 ± 6.7	59.2 ± 6.9	59.5 ± 6.6	59.3 ± 7.0	59.4 ± 6.8	59.7 ± 6.1
Exacerbations in 12 months before study							
0	220 (72)	233 (72)	228 (71)	249 (75)	236 (74)	1,215 (73)	10,021 (61)
1	55 (18)	57 (18)	58 (18)	47 (14)	55 (17)	290 (17)	4,020 (24)
2+	32 (10)	35 (11)	33 (10)	34 (10)	27 (8)	168 (10)	2,444 (15)
Concomitant cardiovascular therapy							
Antiplatelet therapy	176 (57)	196 (60)	197 (62)	231 (70)	238 (75)	1,081 (65)	8,517 (52)
Statin therapy	207 (67)	251 (77)	238 (75)	269 (82)	245 (77)	1,263 (75)	10,721 (65)
Antiplatelet and statin therapy	137 (45)	167 (51)	157 (49)	198 (60)	191 (60)	886 (53)	6151 (37)
Treatment allocation							
Placebo	83 (27)	87 (27)	81 (25)	78 (24)	92 (29)	439 (26)	4,111 (25)
Fluticasone furoate	69 (22)	87 (27)	83 (26)	76 (23)	74 (23)	415 (25)	4,135 (25)
Vilanterol	87 (28)	84 (26)	70 (22)	80 (24)	77 (24)	416 (25)	4,118 (25)
Combination therapy	68 (22)	67 (21)	85 (27)	96 (29)	75 (24)	403 (24)	4,121 (25)

Values are mean ± SD or n (%).

BMI = body mass index; CVD = cardiovascular disease; FEV_1_ = forced expiratory volume in 1 s; GFR = glomerular filtration rate; ITT-E = intention-to-treat efficacy.

∗ Of the 1,673 patients in the biomarker population, 74 did not have baseline cardiac troponin I measured and are therefore not included in the cardiac troponin I quintiles and analyses.

A number of patient characteristics were associated with baseline plasma cardiac troponin I concentration (Table 2). In a multivariate linear regression model, higher baseline plasma cardiac troponin I concentrations were associated with increasing age, male sex, decreased renal function, and other CV risk factors. After adjustment for other variables, higher post-bronchodilator FEV_1_ was associated with lower cardiac troponin I concentrations at baseline.

**Table 2:** Patient Characteristics Associated With Baseline Cardiac Troponin I Concentration

	Univariate Models		Multivariate Model	
	Cardiac Troponin I Ratio	p Value	Cardiac Troponin I Ratio	p Value∗
Age, per 10-yr increase	1.266	<0.001	1.157	<0.001
Male vs. female	1.423	<0.001	1.461	<0.001
BMI, per 5 kg/m^2^ increase	1.043	0.007	1.102	<0.001
Heart rate, per 10 beats/min increase	0.962	0.039	—	—
Systolic blood pressure, per 10 mm Hg increase	1.059	<0.001	1.054	<0.001
Estimated GFR, per 10 ml/min increase	0.974	<0.001	0.965	<0.001
CRP, per 1 mg/l increase	1.005	0.041	—	—
Past medical history vs. absence				
Prior myocardial infarction or coronary revascularization	1.407	<0.001	1.314	<0.001
Coronary artery disease	1.318	<0.001	—	—
Congestive heart failure	1.714	<0.001	1.444	<0.001
Hypercholesterolemia	1.307	<0.001	—	—
Hypertension	1.511	<0.001	1.330	<0.001
Diabetes mellitus	1.114	0.011	—	—
Family history of CVD	1.062	0.158	—	—
Respiratory history				
Smoking status, former smoker vs current smoker	1.098	0.025	0.908	0.019
Post-bronchodilator FEV_1_, per liter increase	0.979	0.642	0.897	0.046
Exacerbations in 12 months before study, vs. 0 exacerbations				
1	0.920	0.135	—	—
2+	0.976	0.730	—	—
Concomitant cardiovascular therapy vs. absence				
Antiplatelet therapy	1.223	<0.001	—	—
Statin therapy	1.121	0.020	—	—

Univariate models contain only that patient characteristic, unadjusted for any other characteristics. Multivariate models also contain all other patient characteristics selected. Cardiac troponin I ratios are expressed relative to a reference, for example, in the univariate model for age, for each 10-year increase, there was a 26.6% increase in concentration; in the univariate model for sex, males had a 42.3% higher concentration than females.

Abbreviations as in Table 1.

∗ p Value after adjusting for all other covariates first.

### Baseline cardiac troponin and risk of clinical events

In the biomarker substudy, during a median on-treatment follow-up of 1.5 (IQR: 0.8 to 2.5) years, there were 74 patients (4.6%) with a composite CV event, and 587 (36.7%) patients with moderate or severe exacerbations of COPD. During a median on- and post-treatment follow-up of 2.3 (IQR: 1.6 to 3.1) years, there were 25 CV deaths.

**Figure 1:**
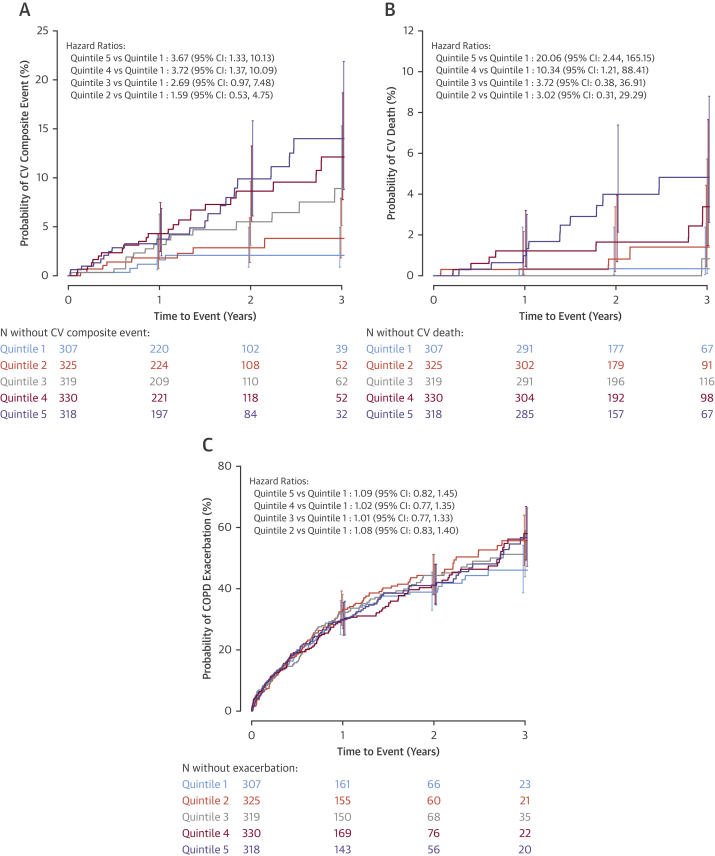
Baseline High-Sensitivity Cardiac Troponin and Risk of CV Composite Events, CV Death, and COPD Exacerbations Patients were grouped into quintiles based on their baseline cardiac troponin I concentrations. Compared with the lowest quintile (<2.3 ng/l), those in the highest quintile (≥7.7 ng/l) were at greater risk of experiencing a CV composite event **(A)** and CV death **(B)**. By contrast, there was no difference between the highest and lowest quintiles in the risk of moderate or severe COPD exacerbations **(C)**. CI = confidence interval; COPD = chronic obstructive pulmonary disease; CV = cardiovascular.

Compared with the lowest quintile, participants in the highest quintile were at greater risk of experiencing a CV composite event (Figure 1A). This difference persisted after adjustment for confounding variables including CV risk factors (Table 3) (hazard ratio [HR]: 3.67; 95% confidence interval [CI]: 1.33 to 10.13; p = 0.012). The association with increased CV risk persisted in the sensitivity analysis with additional adjustment for C-reactive protein and statin therapy (Online Table 1), and was also demonstrated when troponin concentrations were considered in a continuous manner (Central Illustration). Similarly, there was a marked increased risk for CV death in the highest quintile (Table 3) (HR: 20.06; 95% CI: 2.44 to 165.15; p = 0.005) (Figure 1B). By contrast, there was no difference between the highest and lowest quintiles in the risk of moderate or severe COPD exacerbations (Table 3) (HR: 1.09; 95% CI: 0.82 to 1.45; p = 0.548) (Figure 1C).

**Table 3:** Time to First CV Composite Event and Time to CV Death by Baseline Cardiac Troponin Quintiles and Cardiac Troponin I ≥5 ng/l at Either Baseline or 3-Month Time Point

	Troponin Quintile 1 (<2.3 ng/l) (n = 307)	Troponin Quintile 2 (≥2.3 to <3.4 ng/l) (n = 325)	Troponin Quintile 3 (≥3.4 to <4.8 ng/l) (n = 319)	Troponin Quintile 4 (≥4.8 to <7.7 ng/l) (n = 330)	Troponin Quintile 5 (≥7.7 ng/l) (n = 318)
Patients experiencing CV event∗	5 (2)	9 (3)	16 (5)	23 (7)	21 (7)
Quintile vs. 1st quintile					
Hazard ratio†		1.59	2.69	3.72	3.67
95% CI		(0.53–4.75)	(0.97–7.48)	(1.37–10.09)	(1.33–10.13)
p Value		0.409	0.059	0.010	0.012
CV death	1 (<1)	3 (<1)	3 (<1)	7 (2)	11 (3)
Quintile vs. 1st quintile					
Hazard ratio†		3.02	3.72	10.34	20.06
95% CI		(0.31–29.29)	(0.38–36.91)	(1.21–88.41)	(2.44–165.15)
p Value		0.341	0.261	0.033	0.005
Patients experiencing a moderate or severe COPD exacerbation	108 (35)	123 (38)	120 (38)	121 (37)	115 (36)
Quintile vs. 1st quintile					
Hazard ratio‡		1.08	1.01	1.02	1.09
95% CI		(0.83–1.40)	(0.77–1.33)	(0.77–1.35)	(0.82–1.45)
p Value		0.567	0.925	0.886	0.548

	<5 ng/l at Both Time Points (n = 673)	≥5 ng/l at Either Time Point (n = 585)
Patients experiencing CV event∗	22 (3)	42 (7)
≥5 ng/l vs. <5 ng/l		
Hazard ratio†		2.02
95% CI		(1.18–3.46)
p Value		0.011
CV death	3 (<1)	14 (2)
≥5 ng/l vs <5 ng/l		
Hazard ratio†		6.76
95% CI		(1.86–24.56)
p Value		0.004
Patients experiencing a moderate or severe COPD exacerbation	275 (41)	228 (39)
≥5 ng/l vs. <5 ng/l		
Hazard ratio‡		0.94
95% CI		(0.78–1.13)
p Value		0.491

Values are n (%) unless otherwise noted.

CI = confidence interval; COPD = chronic obstructive pulmonary disease; CV = cardiovascular.

∗ Composite CV event comprising any of: CV death, myocardial infarction, stroke, unstable angina, and transient ischemic attack.

† Cox proportional hazards model adjusted for inhaled treatment, age, sex, previous myocardial infarction, hypertension.

‡ Cox proportional hazards model adjusted for inhaled treatment, age, sex, previous myocardial infarction, hypertension, previous COPD exacerbation history.

**Central Illustration:**
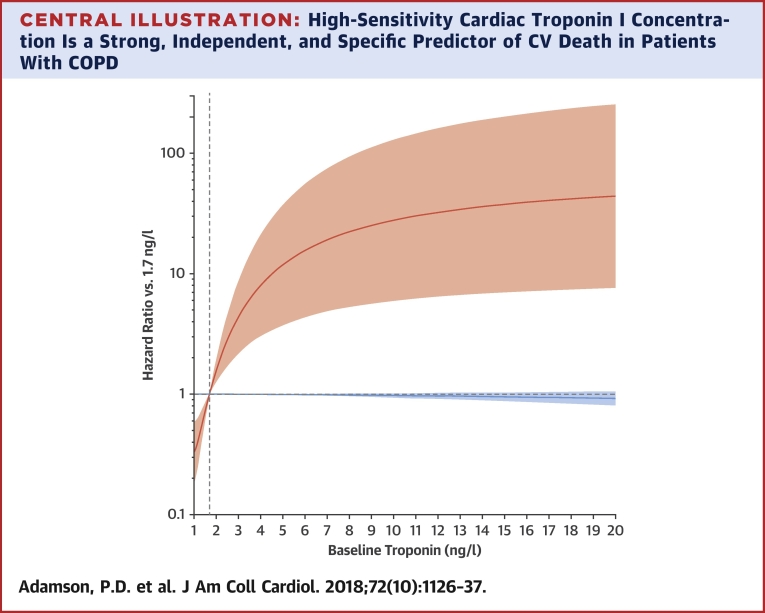
High-Sensitivity Cardiac Troponin I Concentration Is a Strong, Independent, and Specific Predictor of CV Death in Patients With COPD The association between baseline high-sensitivity cardiac troponin I and CV death **(orange)** and COPD exacerbations **(blue)** was examined using cardiac troponin as a continuous variable. Hazard ratios are compared with the median troponin concentration in the first quintile (1.7 ng/l) and are adjusted for age, sex, previous myocardial infarction, hypertension, and exacerbation history. **Shaded areas** represent 95% confidence intervals. COPD = chronic obstructive pulmonary disease; CV = cardiovascular.

### Effect of treatment on cardiac troponin at 3 months

Plasma cardiac troponin I concentrations at 3 months were unchanged from baseline (p > 0.05 for all treatments) (Table 4). There were no treatment-related differences in the change in cardiac troponin I concentration at 3 months (p > 0.05 for all treatments). This was consistent with the lack of treatment effect on the CV composite endpoint (p > 0.05 for all active treatments vs. placebo) (Online Table 2).

**Table 4:** Effect of Inhaled Study Treatment on Cardiac Troponin I Concentration at 3 Months

	Placebo (n = 314)	Fluticasone Furoate 100 (n = 311)	Vilanterol 25 (n = 319)	Fluticasone Furoate/Vilanterol 100/25 (n = 314)
Baseline troponin, ng/l∗	4.4	4.2	4.2	4.0
3-Month troponin, ng/l∗	4.4	4.3	4.1	4.2
Adjusted ratio to baseline† (95% CI)	1.02 (0.96–1.08)	1.02 (0.96–1.08)	0.98 (0.93–1.04)	1.02 (0.97–1.09)
Ratio of 3-month cardiac troponin I in active treatment vs. placebo (95% CI)		1.00 (0.92–1.09)	0.96 (0.89–1.05)	1.01 (0.92–1.09)
p Value		0.947	0.404	0.893

Model is analysis of covariance of log transformed cardiac troponin I, adjusted for baseline cardiac troponin I, age, sex, previous myocardial infarction, and previous hypertension.

CI = confidence interval.

∗ Geometric mean.

† The geometric means displayed are unadjusted, whereas the ratio is based on the model.

### Cardiac troponin threshold and CV events

**Figure 2:**
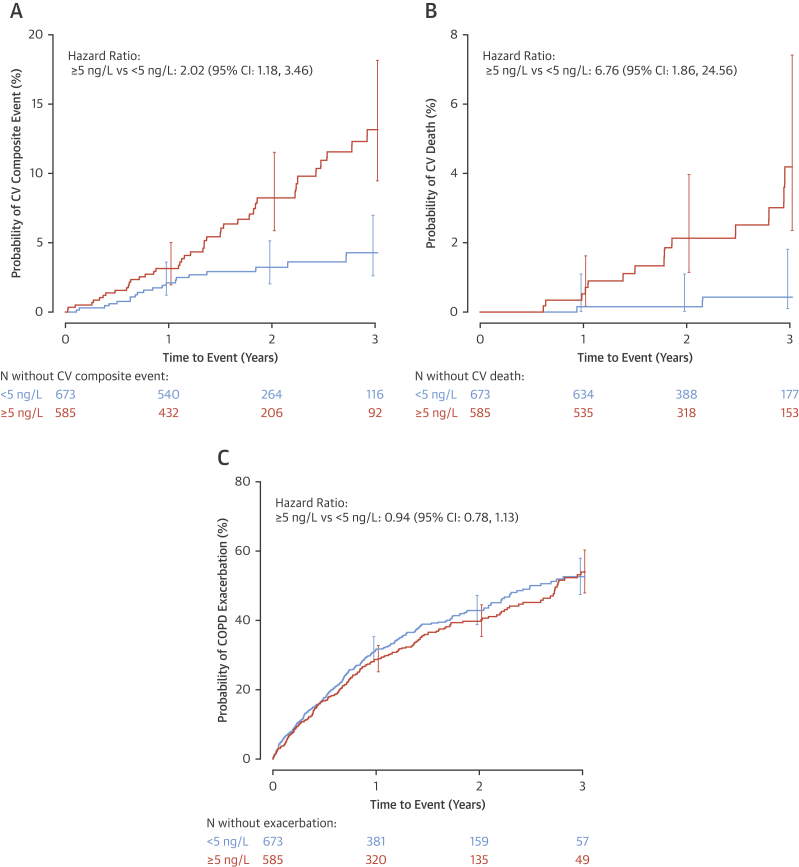
High-Sensitivity Cardiac Troponin at Baseline or 3 Months and Risk of CV Composite Events, CV Death, and COPD Exacerbations Patients were grouped into those who had high-sensitivity cardiac troponin I concentrations <5 ng/l at both baseline and 3 months, and those with a concentration ≥5 ng/l at either baseline or 3 months. Patients with cardiac troponin concentrations ≥5 ng/l at either time point had increased rates of the composite CV endpoint **(A)** and CV death **(B)**. By contrast, there was no difference in COPD exacerbations **(C)**. Abbreviations as in Figure 1.

Of the 1,258 patients with baseline and 3-month measurements, 673 (53%) had cardiac troponin concentrations <5 ng/l on both occasions. Compared with this group, patients who had a plasma troponin ≥5 ng/l at either time point had increased rates of the composite CV endpoint (HR: 2.02; 95% CI: 1.18 to 3.46) and a markedly increased risk of CV death (HR: 6.76; 95% CI: 1.86 to 24.56) (Table 3, Figure 2). By contrast, there was no difference in the endpoint of COPD exacerbations (HR: 0.94; 95% CI: 0.78 to 1.13).

## Discussion

We have identified a strong association between plasma high-sensitivity cardiac troponin I concentration and CV outcomes in patients with COPD at heightened CV risk. Importantly, this relationship was specific for CV events, particularly CV death, with no demonstrable association with the risk of COPD exacerbations despite the apparent association with baseline pulmonary function. Possible mechanisms underpinning this relationship have been described previously and may include increased inflammation, systemic hypoxia, or right ventricular strain associated with pulmonary hypertension 22, 23, 24, 25, 26, 27. In addition, we have shown that the presence of change in troponin concentration on repeat testing over a 3-month period also confers an increased risk of CV events, perhaps reflecting underlying atherosclerotic instability. Furthermore, there was no treatment-related change in plasma troponin concentrations, consistent with the overall neutral effect on all-cause mortality and CV outcomes reported in the primary trial analysis (14).

Our findings highlight the potential use of high-sensitivity cardiac troponin concentration as a surrogate biomarker endpoint in early-phase clinical trials of CV interventions. Our findings also have important clinical implications. Recognizing the risk associated with increased troponin concentrations might encourage clinicians to address CV risk due to lifestyle choices and make patients more likely to engage with these recommendations. Similarly, improved risk stratification may facilitate more appropriate targeting of preventive medications (12). Patients with cardiac troponin concentrations in the upper 2 quintiles are clearly at high risk of CV events, and above the thresholds used in international guidelines for the initiation of lifestyle modification and primary prevention therapies 28, 29, 30. Given that only 60% of these individuals were receiving both antiplatelet and statin therapy, there is a sizeable residual “treatment gap.” By contrast, nearly one-half of the lowest quintile were currently receiving this combination from which they may be deriving limited benefit given their low-risk profile.

This study has a number of notable strengths that distinguish it from previous reports on the use of plasma cardiac troponin within the outpatient setting. First, trial participants comprised a broad spectrum of risk, including primary and secondary prevention populations. Second, as a substudy within the context of a large international randomized trial, we ensured comprehensive follow-up and rigorous adjudication of clinical events. Third, the troponin assay chosen for this analysis is both widely available and analytically robust with <5% of samples below the limit of detection. Finally, the availability of paired plasma samples pre-treatment and after 3 months of therapy allowed assessment of any potential relationship between treatment-related changes in plasma troponin concentration and modification of clinical risk.

Chronic obstructive pulmonary disease is an important risk factor for the onset of CV disease. Although there is a clear correlation between both these conditions and established predisposing factors such as age and smoking history, it appears that the chronic inflammatory milieu that exists in patients with COPD provides additional proatherosclerotic impetus (31). A number of studies have begun to explore this association 32, 33, but questions have persisted regarding the CV safety of inhaled therapies for patients with COPD at increased CV risk 3, 34. The SUMMIT investigators addressed this uncertainty with a large prospective superiority trial using the primary endpoint of all-cause mortality. Notwithstanding its nature as the largest ever randomized placebo controlled trial in the treatment of COPD, the 12% relative reduction in risk was not statistically significant. This major endeavor could perhaps have been avoided had a suitably specific and broadly accepted surrogate indicator of treatment efficacy been available. Candidate biomarkers for the assessment of both pulmonary and CV risk are plentiful; however, most are nonspecific in nature and very few have been demonstrated to hold promise in quantifying treatment efficacy (35). By contrast, cardiac troponin arises solely from the myocardium (36), has consistently demonstrated a strong association with CV outcomes (5), is modifiable with medications (37), and has shown robust correlation between treatment-related concentration change and clinical events 12, 38. When considered alongside this evidence, our findings provide additional support for the hypothesis that plasma cardiac troponin offers a role in the assessment of novel CV interventions and therapies.

Within this study, the troponin concentration that determined the upper quintile was 7.7 ng/l. Compared with the lowest quintile (<2.3 ng/l), the upper quintile was associated with a greater than 3-fold increased risk of all CV events and a 20-fold increased risk of CV death. The application of a troponin concentration threshold of ≥5 ng/l also robustly dichotomized individuals into high- and low-risk groups. This cutpoint of risk is remarkably consistent with previous descriptions 11, 12, 21, 39, 40, 41, and supports the concept of a threshold value above which event rates rise substantially. This threshold is well below the 99th percentile upper reference limit for this assay used for the diagnosis of myocardial infarction, and adoption of troponin testing for CV risk stratification will require additional guidance for clinicians. It is important to note that the distribution of cardiac troponin is highly skewed in reference populations (19), with the upper reference limit increased markedly by a small number of outliers who are likely to have subclinical disease. As such, the cumulative evidence from studies evaluating prognosis rather than diagnosis suggest that the threshold to define low risk (the true normal, perhaps) is at the much lower concentration of 5 ng/l (4). The magnitude of increased risk we identified across troponin quintiles is similar to that seen in both primary and secondary CV disease prevention populations, reinforcing its broad applicability for prognostic stratification 7, 11, 12. Importantly, this threshold is above the 10% coefficient of variation for this assay, and could be used to guide treatment decisions in clinical practice. Our findings have implications for future research. Specifically, risk stratification with high-sensitivity cardiac troponin could be used to more reliably identify and recruit high-risk individuals into pharmacological intervention trials, reducing required sample sizes and avoiding the paradox whereby novel therapies are studied in low-risk populations, but subsequently prescribed for those at much greater risk.

### Study limitations

Our analyses are nonrandomized comparisons, and there is a risk of bias if factors correlated with both troponin and the outcome were not included in our analysis. Due to the additional requirement for specimen collection and storage at baseline and 3 months, this substudy contained only 1,599 (10%) of the total number of participants included in the primary SUMMIT analysis. The baseline characteristics and clinical outcomes appear broadly consistent with the full study analysis although there were some differences in key characteristics such as sex, body mass index, and use of concomitant therapies. In the absence of electrocardiography, we were unable to adjust for the presence of arrhythmia, conduction defects, or left ventricular hypertrophy. Despite employing a robust and precise assay, the average troponin concentrations were low and similar to the concentration where total imprecision of the assay is 10%. This reinforces the need for physicians and trialists to be aware of the analytical characteristics of the locally available troponin assay before implementing cardiac troponin monitoring in clinical practice. Although analytical variation is modest, even at low concentrations 42, 43, when considered at an individual patient level and in combination with any biological variability in patients with COPD, these factors could result in reclassification of risk. Nevertheless, our findings are concordant with previous studies evaluating the prognostic role of high-sensitivity plasma cardiac troponin testing using this assay 11, 12, 21, 39, 40. Although future research studies are required to quantify the effects of analytical and biological variability through repeated sampling in this patient population, misclassification could be addressed in clinical practice through the use of serial troponin measurements over consecutive clinic visits. This would be analogous to the recommendation in clinical guidelines to undertake repeated blood pressure measurements on separate occasions before conferring a diagnosis of hypertension.

## Conclusions

In patients with combined respiratory and CV diseases, high-sensitivity plasma cardiac troponin I concentration is a prognostic marker that is specific to CV, but not respiratory, events. Plasma troponin I concentrations were not modified by the inhaled therapies for COPD investigated in this trial; a finding concordant with the primary SUMMIT findings. As such, high-sensitivity cardiac troponin I represents a plausible surrogate indicator of the CV consequences of novel medical therapies and interventions.

Perspectives **COMPETENCY IN MEDICAL KNOWLEDGE:** In patients with COPD, plasma concentrations of cardiac troponin I are associated with future fatal and nonfatal CV events but not with exacerbations of COPD. Long-acting beta-agonists or ICS, alone or in combination, do not reduce plasma troponin I concentrations or CV events.

**TRANSLATIONAL OUTLOOK:** Serial measurements of high-sensitivity cardiac troponin I could act in future studies as a surrogate market of CV status in patients with COPD and potentially assess the impact of treatment before clinical events occur.
