# Correction

**DOI:** 10.1016/j.jacc.2018.09.031

**Published:** 2018-10-30

Adamson PD, Anderson JA, Brook RD, Calverley PMA, Celli BR, Cowans NJ, Crim C, Dixon IJ, Martinez FJ, Newby DE, Vestbo J, Yates JC, Mills NL

**Cardiac Troponin I and Cardiovascular Risk in Patients With Chronic Obstructive Pulmonary Disease**

**J Am Coll Cardiol 2018;72:1126–37.**

On page 1129, the n values in the table column headings were incorrect.

The corrected Table 1 is shown below:

**Table 1:** Patient Characteristics in the SUMMIT Study Population, the Biomarker Substudy Population, and Split by Cardiac Troponin I Quintile

	Troponin Quintile 1 (<2.3 ng/l) (n = 307)	Troponin Quintile 2 (≥2.3 to <3.4 ng/l) (n = 325)	Troponin Quintile 3 (≥3.4 to <4.8 ng/l) (n = 319)	Troponin Quintile 4 (≥4.8 to <7.7 ng/l) (n = 330)	Troponin Quintile 5 (≥7.7 ng/l) (n = 318)	Biomarker Substudy∗ (n = 1,673)	SUMMIT ITT-E Population (n = 16,485)
Median troponin	1.7	2.8	4.0	5.8	12.0	4.0	-
Age, yrs	63 ± 8	65 ± 8	67 ± 8	68 ± 7	68 ± 7	66 ± 8	65 ± 8
Female	172 (56)	153 (47)	107 (34)	98 (30)	80 (25)	635 (38)	4,196 (25)
BMI, kg/m^2^	30 ± 6	31 ± 7	31 ± 6	31 ± 7	31 ± 7	31 ± 7	28 ± 6
Systolic blood pressure, mm Hg	128 ± 14	129 ± 16	132 ± 16	131 ± 16	134 ± 19	131 ± 16	135 ± 15
Heart rate, beats/min	75 ± 10	74 ± 11	73 ± 11	73 ± 11	73 ± 11	73 ± 11	76 ± 10
Estimated GFR, ml/min/1.73 m^2^	101.2 ± 33.6	100.5 ± 37.1	100.5 ± 38.4	95.2 ± 36.1	89.3 ± 34.9	97.7 ± 36.7	97.3 ± 36.6
CRP, mg/l	5.3 ± 6.9	6.5 ± 8.3	5.6 ± 7.0	6.5 ± 10.3	6.8 ± 8.7	6.2 ± 8.3	6.2 ± 8.3
Past medical history							
Prior myocardial infarction or coronary revascularization	71 (23)	89 (27)	112 (35)	145 (44)	162 (51)	601 (36)	3,436 (21)
Coronary artery disease	113 (37)	132 (41)	148 (46)	186 (56)	199 (63)	818 (49)	8,379 (51)
Congestive heart failure	15 (5)	11 (3)	21 (7)	34 (10)	61 (19)	146 (9)	3,456 (21)
Hypercholesterolemia	243 (79)	280 (86)	283 (89)	295 (89)	292 (92)	1458 (87)	11,518 (70)
Hypertension	258 (84)	285 (88)	300 (94)	307 (93)	302 (95)	1519 (91)	14,851 (90)
Diabetes mellitus	108 (35)	116 (36)	116 (36)	138 (42)	140 (44)	642 (38)	4,997 (30)
Family history of CVD	128 (42)	128 (39)	116 (36)	146 (44)	145 (46)	691 (41)	3,429 (21)
Respiratory history							
Former smoker	141 (46)	152 (47)	170 (53)	178 (54)	169 (53)	845 (51)	8,807 (53)
Post-bronchodilator FEV_1_, l	1.7 ± 0.4	1.7 ± 0.4	1.7 ± 0.4	1.7 ± 0.4	1.7 ± 0.4	1.7 ± 0.4	1.7 ± 0.4
Predicted post-bronchodilator FEV_1_, % of predicted	59.7 ± 6.9	59.4 ± 6.7	59.2 ± 6.9	59.5 ± 6.6	59.3 ± 7.0	59.4 ± 6.8	59.7 ± 6.1
Exacerbations in 12 months before study							
0	220 (72)	233 (72)	228 (71)	249 (75)	236 (74)	1,215 (73)	10,021 (61)
1	55 (18)	57 (18)	58 (18)	47 (14)	55 (17)	290 (17)	4,020 (24)
2+	32 (10)	35 (11)	33 (10)	34 (10)	27 (8)	168 (10)	2,444 (15)
Concomitant cardiovascular therapy							
Antiplatelet therapy	176 (57)	196 (60)	197 (62)	231 (70)	238 (75)	1,081 (65)	8,517 (52)
Statin therapy	207 (67)	251 (77)	238 (75)	269 (82)	245 (77)	1,263 (75)	10,721 (65)
Antiplatelet and statin therapy	137 (45)	167 (51)	157 (49)	198 (60)	191 (60)	886 (53)	6151 (37)
Treatment allocation							
Placebo	83 (27)	87 (27)	81 (25)	78 (24)	92 (29)	439 (26)	4,111 (25)
Fluticasone furoate	69 (22)	87 (27)	83 (26)	76 (23)	74 (23)	415 (25)	4,135 (25)
Vilanterol	87 (28)	84 (26)	70 (22)	80 (24)	77 (24)	416 (25)	4,118 (25)
Combination therapy	68 (22)	67 (21)	85 (27)	96 (29)	75 (24)	403 (24)	4,121 (25)

Values are mean ± SD or n (%).

BMI = body mass index; CVD = cardiovascular disease; FEV_1_ = forced expiratory volume in 1 s; GFR = glomerular filtration rate; ITT-E = intention-to-treat efficacy.

∗ Of the 1,673 patients in the biomarker population, 74 did not have baseline cardiac troponin I measured and are therefore not included in the cardiac troponin I quintiles and analyses.

The authors apologize for these errors.

The online version of the article has been corrected to reflect these changes.

