# Supplementary material for: Cardiac Troponin I and Cardiovascular Risk in Patients With Chronic Obstructive Pulmonary Disease
Source: J Am Coll Cardiol. 2018 Sep 4;72(10):1126–37. doi: 10.1016/j.jacc.2018.06.051 (PMC6119211; doi:10.1016/j.jacc.2018.06.051)
Supplement: Online Tables 1 and 2 [file mmc1.docx]

**SUPPLEMENT**

**Cardiac troponin I and risk of cardiovascular events in patients with COPD and heightened cardiovascular risk**

Philip D Adamson^1^, Julie A Anderson^2^, Robert D Brook^3^, Peter MA Calverley^4^, Bartolome R Celli^5^, Nicholas J Cowans^6^, Courtney Crim^7^, Ian J Dixon^6^, Fernando J. Martinez^8^, David E Newby^1^, Jørgen Vestbo^9^, Julie C Yates^7^, Nicholas L Mills^1^

^1^British Heart Foundation Centre for Cardiovascular Science, University of Edinburgh, Edinburgh, UK; ^2^Research & Development, GSK, Stockley Park, Middlesex, UK; ^3^Division of Cardiovascular Medicine, University of Michigan, Ann Arbor, MI, USA; ^4^Department of Medicine, Clinical Sciences Centre, University of Liverpool, University Hospital Aintree, Liverpool, UK; ^5^Pulmonary and Critical Care Division, Brigham and Women’s Hospital, Harvard Medical School, Boston, MA, USA; ^6^Statistics and Programming, Veramed Ltd, Twickenham, UK; ^7^Research & Development, GSK, Research Triangle Park, NC, US; ^8^Joan and Sanford I. Weill Department of Medicine, Weill Cornell Medicine, New York, NY, USA; ^9^Division of Infection, Immunity and Centre for Respiratory Medicine and Allergy, Manchester Academic Health Science Centre, The University of Manchester and Manchester University NHS Foundation Trust, Manchester, UK;

**Correspondence and requests for reprints:**

Professor Nicholas L Mills

BHF/University Centre for Cardiovascular Science

The University of Edinburgh

Edinburgh EH16 4SA

United Kingdom

Email: [nick.mills@ed.ac.uk](mailto:nick.mills@ed.ac.uk)

**Supplementary Table 1.** Time to first cardiovascular composite event and time to cardiovascular death by baseline cardiac troponin quintiles

|  | **Troponin Quintile 1 (<2.3 ng/L) (n=307)** | **Troponin Quintile 2 (>=2.3 to <3.4 ng/L) (n=325)** | **Troponin Quintile 3 (>=3.4 to <4.8 ng/L) (n=319)** | **Troponin Quintile 4 (>=4.8 to <7.7 ng/L) (n=330)** | **Troponin Quintile 5 (>=7.7 ng/L) (n=318)** |
| --- | --- | --- | --- | --- | --- |
| Number in analysis^¥^ | 305 | 323 | 316 | 326 | 318 |
|  |  |  |  |  |  |
| Patients experiencing CV event* | 5 (2%) | 9 (3%) | 16 (5%) | 23 (7%) | 21 (7%) |
| Quintile vs. 1st quintile |  |  |  |  |  |
| Hazard ratio^ |  | 1.57 | 2.76 | 3.68 | 3.69 |
| 95% CI |  | (0.52, 4.69) | (0.99, 7.66) | (1.36, 9.97) | (1.34, 10.19) |
| p-value |  | 0.423 | 0.052 | 0.010 | 0.012 |
|  |  |  |  |  |  |
| CV death | 1 (<1%) | 3 (<1%) | 3 (<1%) | 7 (2%) | 11 (3%) |
| Quintile vs. 1st quintile |  |  |  |  |  |
| Hazard ratio^ |  | 3.19 | 3.77 | 11.23 | 21.00 |
| 95% CI |  | (0.33, 31.06) | (0.38, 37.40) | (1.30, 96.60) | (2.53, 174.18) |
| p-value |  | 0.318 | 0.257 | 0.028 | 0.005 |
|  |  |  |  |  |  |
| Patients experiencing a moderate or severe COPD exacerbation | 108 (35%) | 122 (38%) | 119 (38%) | 121 (37%) | 115 (36%) |
| Quintile vs. 1st quintile |  |  |  |  |  |
| Hazard ratio§ |  | 1.08 | 1.01 | 1.03 | 1.09 |
| 95% CI |  | (0.83, 1.40) | (0.77, 1.33) | (0.78, 1.36) | (0.82, 1.45) |
| p-value |  | 0.582 | 0.932 | 0.840 | 0.559 |

¥ 11 patients did not have baseline CRP so could not be included in the analysis.

*Composite cardiovascular event comprising any of: cardiovascular death, myocardial infarction, stroke, unstable angina and transient ischemic attack.

CV, cardiovascular; CI, confidence intervals

^Cox proportional hazards model adjusted for inhaled treatment, age, sex, previous MI, hypertension, CRP and statin use

§Cox proportional hazards model adjusted for inhaled treatment, age, sex, previous MI, hypertension, previous COPD exacerbation history, CRP and statin use

**Supplementary Table 2.** Effect of inhaled therapies on cardiovascular composite endpoint in the SUMMIT biomarker population

|  | **Placebo (N=439)** | **FF 100 (N=415)** | **VI 25 (N=416)** | **FF/VI 100/25 (N=403)** |
| --- | --- | --- | --- | --- |
| n with baseline Troponin | 421 | 389 | 398 | 391 |
|  |  |  |  |  |
| Patients experiencing a CV Event | 20 (5%) | 20 (5%) | 19 (5%) | 15 (4%) |
| Active vs. Placebo |  |  |  |  |
| Hazard Ratio |  | 0.89 | 0.91 | 0.67 |
| 95% CI |  | (0.48, 1.67) | (0.49, 1.72) | (0.34, 1.32) |
| p-value |  | 0.723 | 0.778 | 0.242 |

Cox proportional hazard model adjusted for baseline cardiac troponin I tertiles, age, gender, previous MI and previous hypertension.
